# Mapping global Muslim mental health research: analysis of trends in the English literature from 2000 to 2015

**DOI:** 10.1017/gmh.2019.3

**Published:** 2019-05-16

**Authors:** H. H. Altalib, K. Elzamzamy, M. Fattah, S. S. Ali, R. Awaad

**Affiliations:** 1 Departments of Neurology and Psychiatry, Yale University School of Medicine, New Haven, CT, USA; 2 VA Connecticut Health System West Haven Campus, West Haven, CT 06516, USA; 3 Department of Psychiatry, Hamad Medical Corporation, Doha, Qatar; 4 Department of Psychiatry, Stanford Hospital and Clinics, Stanford, CA, USA; 5 Department of Psychiatry, University of New Mexico, Albuquerque, New Mexico, USA

**Keywords:** Bibliometric analysis, mental Health, Muslims, network analysis, Islam

## Abstract

**Background.:**

By 2030, the global Muslim population is expected to reach 2.2 billion people. The representations of Islam and Muslims in the media and academic literature may unconsciously impact how clinicians perceive and approach their Muslim patients. Our study focuses on the emerging Muslim mental health (MMH) literature using bibliometric analysis, specifically social network analysis of word co-occurrence and co-authorship networks of academic publications, to describe how the content of MMH discourse is evolving.

**Methods.:**

We conducted an Ovid search (including Medline and PsycInfo databases) to identify articles written in English from 2000 to 2015 that had the terms ‘Islam’ and/or ‘Muslim’ in the abstract as well as research conducted in Muslim-majority countries and among Muslim minorities in the rest of the world.

**Results.:**

Of the 2652 articles on MMH, the majority (65.6%) focused on describing psychopathology; the minority (11.2%) focused on issues around stigma, religiosity, spirituality, identity, or acculturation. Among the top 15 most frequent terms in abstracts were ‘post-traumatic stress disorder’, ‘violence’, ‘fear’, ‘trauma’, and ‘war’. Social network analysis showed there was little collaborative work across regions.

**Conclusions.:**

The challenges of producing MMH research are similar to the challenges faced across global mental health research. Much of the MMH research reflects regional challenges such as the impact of conflict and violence on mental health. Continued efforts to develop global mental health researchers through cross-cultural exchanges, academic journals' dedicated sections and programs for global mental health recruitment, and online training are needed to address the gap in research and collaborations.

## Background

The estimated 1.6 billion Muslims worldwide are expected to grow to 2.2 billion by 2030 (Grim & Hsu, 2011). Clinicians will likely provide care for a Muslim some time in their careers and, depending on the location of their practice, may deliver care to a relatively large and diverse group of Muslims. Against the backdrop of recent national and international political events, more attention has been drawn to the mental health needs of Muslims worldwide as a result of the increasing incidents of discrimination and violence against Muslims, immigration problems, refugee resettlement, and asylum crisis (Ahmed & Reddy, 2007; Oppedal & Røysamb, 2007; Khan & Khan, 2013).

These factors have resulted in an increasing demand to understand the mental health needs of Muslims with a growing focus on research and publications. The increased interest in Muslim mental health (MMH) is evidenced by the increase in the number of books (Dwairy, 2006; Ahmed & Amer, 2012; Daneshpour, 2017), research centers, and peer-reviewed articles that invoke the terms ‘Muslim’ or ‘Islam’. Textbooks such as ‘Counseling Muslims’ (Ahmed & Amer, 2012), ‘Family Therapy with Muslims’ (Daneshpour, 2017), and ‘Counseling and Psychotherapy with Arabs and Muslims’ (Dwairy, 2006) have been published by large, mainstream publishing houses for readers interested in delivering mental health services (MHS) to those who identify as (or are identified as) Muslims.

Traditionally, the study of mental health and culture is framed around ethnicity, nationality, and race. However, an emerging literature describing the mental health of ‘Muslim’ populations suggests religiosity as a point of interest. The common theme of the MMH literature is that Islam as a religion often informs how emotional distress is conceptualized and expressed, shapes interpersonal roles and relationships, and impacts health-seeking behavior in Muslim subcultures (Cinnirella & Loewenthal, 1999; Taylor *et al*. 2000; Abdel-Khalek, 2011; Padela *et al*. 2012).

Despite the growing body of literature studying Muslims, no review has yet characterized the publications produced, types of research being conducted, and research gaps that exist. Since Muslim populations are distributed globally, beyond Muslim-majority countries, examining the effect of geographical resources on research productivity and the scope of collaborations among researchers across regions will inform how research is constructed. Understanding trends in the MMH literature provides valuable information to clinicians, researchers, public health providers, policy makers, and other key stakeholders interested in delivering mental health care to Muslim populations and diaspora communities. Furthermore, by understanding how the MMH literature is constructed, the inherent biases and subjectivities of the literature will help readers and stakeholders contextualize MMH research.

Studying the evolution of MMH discourse is undertaken in two steps. First, this study describes the content and themes of the MMH literature. Second, this study analyzes patterns of international collaborations among countries producing research on MMH using bibliometric analysis. Bibliometric analysis is a research methodology that quantifies how content (co-word occurrence) and researcher (co-authorship) networks are connected (Ellegaard & Wallin, 2015). Traditional methods of systematically reviewing a literature begin with identifying a research question or topic (oftentimes related to a clinical intervention); selecting the most relevant research articles that address the research question/topic; then summarizing the results, including potential biases and limitations, of the studies. Instead of beginning with an *a priori* research topic or question, bibliometric analysis provides insights into the characteristics of MMH research outputs by quantifying key themes and key concepts, growth in the type of publications, citation patterns, how information is organized, and how disciplines are connected. This approach also helps with identifying research trends and gaps that can guide new research projects. Examination of researcher networks may also provide insight into who and how the research literature is being shaped.

## Methods

### Overview of the bibliometric analysis method

Bibliometric analysis encompasses various tools, including network analysis, to quantify connections across a literature. For instance, word co-occurrence uses metadata from manuscripts, such as the keywords or text of an abstract, and counts how many times similar words cluster across manuscripts. Patterns in how terms cluster together provide a sense of how meaning is constructed. Another tool of bibliometric analysis is mapping co-authorship networks. The number of articles two authors publish together reflects the extent to which scientific collaborations occur. The characteristics of the authors and their networks may inform how the literature in a particular field is shaped, as well as where future collaborations are more likely to occur.

### Search strategy and selection criteria

#### Defining the scope of Muslim mental health

Articles related to Islam or the Muslim population and mental health were extracted through Ovid (including Medline and PsycInfo databases). Mental health was defined by the following subject search: (‘Mental disorders’[subject] OR ‘Mental health’[subject]). Muslims and Islam were operationalized either by keyword searches (‘Muslims’ OR ‘Islam’) or by populations from Muslim-majority countries [as defined by >50% of the population of the country being Muslim (Pew Research Center, 2017)]. People from Muslim-majority countries were identified by keyword search using the stem names of the countries (for instance, ‘Morroc’ for ‘Morroco’ or ‘Morrocan’ (see Appendix)). This technique allows for identifying studies conducted on Muslim populations within their home countries as well as the Muslim diaspora. For instance, studies on attitudes toward MHS among African-American Muslims in the USA, prevalence of depression among Syrian refugees in France, and incidence of suicide in Pakistan would all be captured using the above search terms.

#### Article categorization

Only journal articles published in English, conducted on humans, and indexed in PsycINFO or Medline between the years 2000 and 2015 were included. Abstract collections, bibliographies, opinion articles, editorials, errata, letters, obituaries, and poetry were all excluded. We also excluded publication information, reprints, and reviews (including book reviews, media reviews, software reviews, and other reviews).

The primary reviewer (KE) with mental health expertise screened the titles and abstracts, and identified all potentially eligible articles. The categorization of articles was reviewed and adjudicated by the corresponding author (HA). The primary reviewer manually excluded search results that were not original research articles, not studying Muslim populations and/or Islam, or not related to mental health. Articles excluded fell into four categories: (1) books, handbooks, book sections, and book chapters; (2) case studies and case reports; (3) clinical trials that did not clearly include Muslim majority countrie;s and (4) studies on military and veteran studies of non-Muslim-majority countries (such as the USA or UK) that operated in Muslim countries such as Iraq and Afghanistan. If a duplicate of an article was found, only one copy was retained.

Based on the titles and abstracts, we categorized articles into eight groups initially: (1) general mental health and well-being; (2) psychopathology and mental disorders; (3) substance abuse; (4) MHS; (5) stigma and attitude; (6) family, marriage, and sexuality; (7) religiosity and spirituality; and (8) identity and acculturation. For articles that studied more than one mental disorder, a copy was placed under each category. The main themes of articles included under each category were identified and documented (Table 1).

**Table 1.** Count of Muslim mental health articles based on topic category

	Count
Psychopathology	
Mood disorders/suicide	576
Anxiety/OCD/PTSD/trauma-related	445
Schizophrenia/psychotic disorders	226
Childhood and infancy disorders	111
Delirium/dementia disorders	105
Substance use disorders	81
Personality traits and personality disorders	46
Eating disorders	45
Dissociative disorders	25
Somatoform disorder	18
Abuse and neglect/other disorders	14
Sexual and gender disorders	12
Sleep disorders	9
Impulse control disorders	5
Adjustment disorders	5
Muslim mental health topics	
General MH, well-being and disorders/QOL	484
Mental health services	251
Stigma/attitudes	157
Religiosity/spirituality	111
Family/marriage/sexuality	76
Identity/acculturation	30

#### Author identification

All authors were manually reviewed to prevent duplicates based on Scopus profile. Authors who published at least two articles in the MMH field were included. Authors' gender that was not easily identifiable was checked through a Google search and/or run through Gender API (https://gender-api.com/). Author country and academic affiliation were identified by cross-matching with authors' Scopus profiles. This information was extracted using Elsevier's Scopus API (https://dev.elsevier.com/). For cases in which the same author had multiple profiles, the country with the greatest number of publications or most recent affiliation was chosen. Authors who published at least two articles with the term ‘Muslim’ or ‘Islam’ in the title and/or abstract were identified as having published directly on the topic.

#### Bibliometric analysis

The MMH co-authorship network was extracted using the Science of Science tool (Sci2 Team, 2015) and was calculated and visualized using Gephi (Bastian *et al*. 2009) with nodes representing individual authors and edges reflecting the number of publications co-authored between two authors. Standard measures of network centrality as well as whole-network measures were calculated as follows (Borgatti *et al*. 2013):

*Network density*: number of existing ties between any two authors in relation to the number of all possible links between all authors within the network.

*Node degree*: number of ties an author has with other authors.

*Betweenness centrality*: the extent to which an author connects other authors together or, in other words, how often lies along the shortest path between all other authors within the network.

*Closeness centrality*: how close, with respect to degrees of separation, an author is from all other authors of the network, calculated by summing the author of interest and the shortest paths of all authors to her.

Word co-occurrence networks were extracted using VOSviewer (Van Eck & Waltman, 2009, 2014). The term-mining, natural language processing, and visualization techniques of the software are detailed elsewhere (Van Eck & Waltman, 2009). Briefly, nouns within abstracts are extracted and linked by distance to other nouns. The terms ‘ptsd’ and ‘post-traumatic stress disorder’ were joined because they are synonymous concepts; ‘trauma’ and ‘traumatic event’ were combined to avoid redundancy; and ‘way’ (observed 51 times in 2011–2015) was removed since it did not appear relevant. Multidimensional scaling is used to map the geometric distance of each term relates to one each other. For the purposes of this analysis, terms from abstracts of MMH articles were analyzed together across the whole time span of 2000–2015 as well as in 5-year blocks. Binary counting was chosen such that if a word occurred several times within the title and/or abstract it would only be counted once.

## Results

The search generated 5526 articles based on our search criteria after a manual review of the articles. After excluding articles related to clinical trials of psychotropic medications, non-mental health articles, US/Great Britain/Australia veteran or military mental health serving in Iraq or Afghanistan, and articles in which ‘Islam’ or ‘Muslim’ was the name of an author and not a concept in the abstract or title, 2744 articles remained. The primary categories of articles published are shown in Table 1 (88 articles fell into two categories).

Of the 2744 articles identified, 2218 articles primarily explore mental health issues in Muslim-majority populations; 374 articles were focused on Muslim diaspora, refugee, and/or minority populations; and 152 articles were related to multi-site, multiple country studies. Most MMH research published describes psychopathology (Table 1). Original research on the role of Islam and Muslim culture on the expression of and coping with emotional distress constitutes <5% of the MMH literature and is primarily published by researchers based in medium- and high-income Muslim-majority countries such as Turkey, Iran, and Malaysia.

### MMH research themes and geographical productivity

**Fig. 1.**
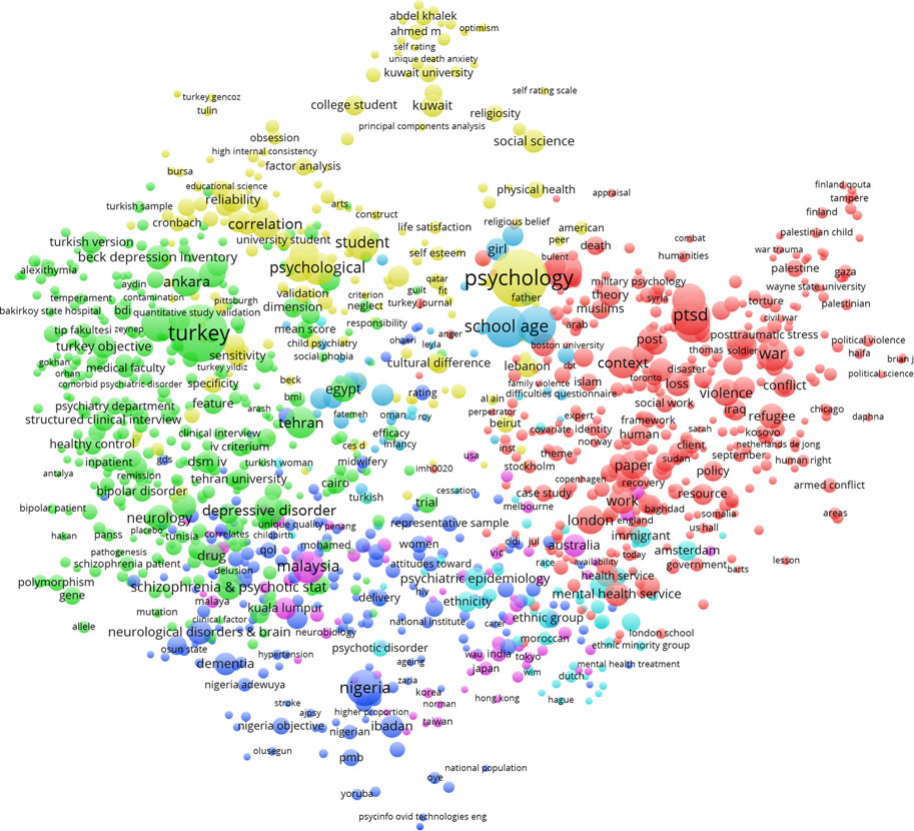
Word co-occurrence network within the Muslim mental health research discourse.

Word co-occurrence, which maps key words or words within and across abstracts of publications, reflects the connections between themes across the literature (van Eck & Waltman, 2014). Table 3 includes the 15 most common terms in the literature during the respective time frame. Word co-occurrence analysis demonstrated how common (Table 3) and central (Fig. 1) constructs such as ‘fear’, ‘trauma’, ‘violence’, ‘war’, and ‘post-traumatic stress’ were to the MMH literature.

### Results of the co-authorship networks

**Fig. 2.**
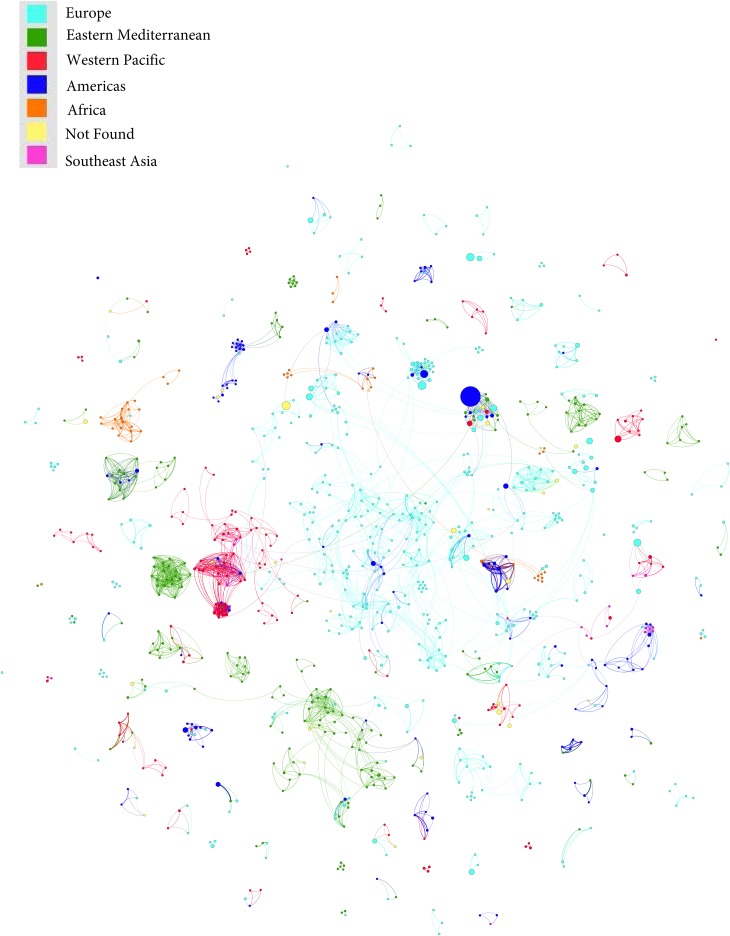
Co-authorship networks of Muslim mental health researchers.

Co-authorship networks map how researchers are connected to each other and provide a social context to how a literature develops (Fig. 2). Each circle (or node) within Fig. 2 represents an individual author. Nodes are connected (by curved lines or edges) if two authors co-authored at least one paper together. Nodes and authors are colored according to the region, as defined by the World Health Organization (WHO), of their primary academic affiliation. For instance, the green clusters represent the Eastern Mediterranean (primarily the Middle East) and often publish only with authors from the same region and occasionally with authors from North America, Europe, and the Western Pacific.

MMH articles included in the network analysis were written by 8140 co-authors. Only 1042 corresponding authors published at least two articles on MMH and only 69 authors of those specifically wrote about Islam and/or Muslims. The majority of corresponding authors are from Turkey (*n* = 243), the USA (*n* = 115), Iran (*n* = 78), Malaysia (*n* = 60), the Netherlands (*n* = 57), Egypt (*n* = 51), the UK (*n* = 49), and Nigeria (*n* = 45). However, authors from North America and Western Europe are more prolific (Table 2).

**Table 2.** Top 10 most cited and published authors

Gender	Citation count	Cited-by-count	Country	Coauthor count	*H*-index	Number of Publications	Degree	Closeness	Betweeness	Triangles	Eigencetrality
**a) Top 10 most cited authors**											
M	15436	9335	United States	524	67	421	4	0.1461	0.0000	6	0.0071
M	13831	9799	United States	150	34	1985	3	1.0000	0.0000	3	0.0027
M	12758	7203	United States	507	56	355	2	1.0000	0.0000	1	0.0015
M	11610	9684	United Kingdom	150	56	426	2	0.1413	0.0000	1	0.0029
M	7251	5877	United Kingdom	376	45	191	2	1.0000	0.0000	1	0.0015
M	3908	2509	Australia	205	36	98	6	0.1462	4.5000	9	0.0094
M	3354	2676	United States	194	32	151	10	0.5625	91.6476	25	0.0269
M	2710	2026	United States	117	25	130	3	1.0000	0.5000	2	0.0024
M	2622	1969	Canada	104	19	58	2	0.0974	0.0000	1	0.0020
M	2542	2005	Singapore	150	25	60	10	0.1814	308.8597	42	0.0871
**b) Top 10 most cited authors from Muslim majority countries**											
M	2038	1006	Egypt	129	23	159	3	1.0000	0.0000	3	0.0027
M	1982	1515	Iran	394	25	162	16	0.3482	430.2447	60	0.0847
M	1061	867	Oman	238	17	106	13	0.9333	42.3667	30	0.0233
M	982	900	Morocco	316	16	79	2	0.1144	0.0000	1	0.0020
M	751	704	Turkey	66	16	62	8	0.1370	6037.7667	4	0.0084
M	678	561	Turkey	75	13	68	7	0.4524	78.0000	11	0.0105
F	625	598	Turkey	75	13	30	7	0.1537	15706.7668	6	0.0341
M	600	579	Morocco	215	13	33	5	0.1291	1110.0000	4	0.0049
M	429	262	Iran	40	12	40	3	1.0000	0.5000	2	0.0024
M	375	346	Turkey	37	8	16	7	0.1762	2212.0000	9	0.0156
**c) Top 10 most published authors**											
M	13831	9799	United States	150	34	1985	3	1.0000	0.0000	3	0.0027
M	11610	9684	United Kingdom	150	56	426	2	0.1413	0.0000	1	0.0029
M	15436	9335	Australia	524	67	421	4	0.1461	0.0000	6	0.0071
M	12758	7203	United States	507	56	355	2	1.0000	0.0000	1	0.0015
M	7251	5877	United Kingdom	376	45	191	2	1.0000	0.0000	1	0.0015
M	1982	1515	Iran	394	25	162	16	0.3482	430.2447	60	0.0847
M	2038	1006	Egypt	129	23	159	3	1.0000	0.0000	3	0.0027
M	3354	2676	United States	194	32	151	10	0.5625	91.6476	25	0.0269
M	2710	2026	United States	117	25	130	3	1.0000	0.5000	2	0.0024
F	1899	1514	United States	500	24	124	3	0.5385	12.5000	0	0.0023
**d) Top 10 most published authors from Muslim majority countries**											
M	1982	1515	Iran	394	25	162	16	0.3482	430.2447	60	0.0847
M	2038	1006	Egypt	129	23	159	3	1.0000	0.0000	3	0.0027
M	1061	867	Oman	238	17	106	13	0.9333	42.3667	30	0.0233
M	982	900	Morocco	316	16	79	2	0.1144	0.0000	1	0.0020
M	678	561	Turkey	75	13	68	7	0.4524	78.0000	11	0.0105
F	751	704	Turkey	66	16	62	8	0.1370	6037.7667	4	0.0084
M	291	253	Oman	149	10	49	8	0.7000	7.8333	18	0.0162
F	291	278	Malaysia	83	9	41	6	0.1293	2259.3778	3	0.0072
M	429	262	Iran	40	12	40	3	1.0000	0.5000	2	0.0024
M	600	579	Morocco	215	13	33	5	0.1291	1110.0000	4	0.0049

Table 2 summarizes author demographic, publication record, and network analysis results.

## Discussion

The volume of MMH research is grossly disproportionate to the global Muslim population. For instance, the total number of MMH articles globally is less than the number of mental health publications of many individual academic institutions in the USA and UK during the same period (and not related to MMH). One possible hypothesis for the disparity in publications is directly related to the limited size of the mental health workforce; however, this requires further research. The shortage of the global mental health workforce and its impact on research is well described (Kakuma *et al*. 2011).

A large proportion of researchers who published in the MMH literature were from Muslim-majority countries (Turkey, Iran, Malaysia, Nigeria, and Egypt). We expected, much like the general trend in the global mental health literature, that North American and Western European researchers would be most prolific (Bouchard *et al*. 2015). A bibliometric analysis of mental health research from 1980 to 2011 by RAND demonstrated that 95% of mental health research was produced by 20 countries; however, in more recent years, countries such as Brazil, Russia, India, and China have increased their relative proportion of publications (Larivière *et al*. 2013). Even within the cross-cultural mental health literature, the USA publishes over 50% of articles in the field, despite eastern countries being better represented (Allik, 2013). In contrast, the MMH literature has many more authors publishing from Muslim-majority countries compared with the general global mental health literature. Perhaps the efforts made by the WHO and other global mental health stakeholders to close the gap in mental health research are paying off. However, it should be noted that the three most prolific countries (Turkey, Iran, and Malaysia) are higher-income countries. Further work is needed to help support the research in low-income Muslim-majority countries. In spite of the productivity of researchers from Muslim-majority countries, the top 10 most cited authors as well as published researchers were based in the USA, the Netherlands, and Australia. All of these researchers work on large international epidemiologic programs and much of their work is not related directly to MMH.

Many of the researchers most central (as measured by betweenness centrality) to the MMH scholarly network were from higher income, Muslim-majority countries such as Turkey, Iran, and Malaysia; this suggests that they may be more collaborative and may have more potential to connect scholars across different networks. However, they appear to primarily connect with authors from their own region. Recently, there has been an increased proportion of international collaborations across global mental health research (from 3% in 1980 to 19% in 2008) (Larivière *et al*. 2013); however, the overwhelming majority of these collaborations are between North American and Western European countries. In contrast, MMH research collaborations tend to be insular with little collaboration between Muslim-majority countries and North America or Europe.

As for MMH research themes, topics related to ‘trauma’, ‘violence’, ‘war’, and ‘post-traumatic stress’ were the most prominent (Table 3). Other common psychiatric topics such as generalized anxiety disorders, substance use, and psychotic disorders were not prominently represented across regions. By contrast, in the more general mental health literature, substance abuse, depression, anxiety, psychotic disorders, and dementia are prominently featured across countries' mental health research.

In the MMH network, there appeared to be regional differences. For instance, Iraq and Palestine tend to publish more on trauma, war, violence, and conflict; European countries tend to focus on refugees and access to health services; and Nigeria tends to focus on dementia and schizophrenia.

**Table 3.** Most common terms in Muslim mental health research abstracts

2001–2005 occurrences		2006–2010 occurrences		2011–2015 occurrences	
Scale	90	ptsd/posttraumatic stress disorder	155	Trauma/traumatic event	128
ptsd/posttraumatic stress disorder	90	Exposure	70	War	89
correlation	38	Article	67	Exposure	81
Trauma	36	War	64	Validity	76
Time	35	Paper	62	Subscale	74
Self	32	Depressive disorder	60	Paper	73
Item	31	Validity	59	Health service	63
Validity	29	Correlation	50	Effectiveness	58
Exposure	29	Trauma	47	Conflict	54
Depression scale	28	Ratio	46	Dementia	53
War	27	Reliability	45	Beck depression inventory	49
Fear	26	Member	44	Review	49
Health problems	26	Violence	42	Psychological distress	47
Society	25	Understanding	41	dsm iv	46
Anxiety disorder	25	Girl	40	Iraq	42

The major risk of insular networks that lack researchers from Muslim-majority countries is the misrepresentation of the social and cultural context of the region. While violence, war, and conflict are extremely important social forces that inevitably impact the mental health context of a community, they do not sufficiently account for the expansive mental health needs of any one community. This is likely best illustrated in post-conflict, low-income countries where researchers have focused largely on symptoms rather than on full psychiatric diagnostic assessments, thus leading to skewed results that show PTSD and anxiety disorders being the most common post-conflict psychiatric sequelae. The concern with this is that ensuing post-conflict mental health programs may then more heavily focus their efforts on PTSD rather than on common psychiatric conditions.

Currently, mental health efforts in the developing world are based primarily on evidence from high-income countries. This approach has a serious disadvantage for Muslim-majority countries – many of which are considered low- or middle-income countries – in that the majority of the available information about mental health is collected in vastly different cultural and socio-economic contexts. Culturally relevant research should inform mental health policy and service development, treatment decision-making, and anti-stigma and discrimination programs. Yet, this is not possible when developing countries spend <1% of their limited health budgets on mental health (WHO, 2011).

The limitations of this review include a lack of access to detailed information about the authors. Factors such as age, where authors trained and worked, and migrated would have been useful, but were not available. Another limitation of this analysis is that only the English literature was extracted; therefore, regional articles published in Arabic, Farsi, Malay, and other languages that may focus on MMH research were excluded. Much of the research in Muslim-majority countries was focused on basic epidemiologic and psychopathology questions, not specifically on the role of culture, religion, or spirituality on the participants of interest. Therefore, many of the important scholars (and authors) may have no particular interest in MMH, but, because of their role as consultants and/or senior investigators, may have been co-authors on papers in Muslim-majority countries; this is the primary reason that we conducted a separate analysis on papers specifically related to Islam and Muslim culture. Furthermore, Muslims are extremely diverse, especially in the global context, and should not be essentialized and conflated into a single monolithic group. This bibliometric analysis provides a general review of how Muslims across regions are represented in the mental healthcare literature.

Further research in basic mental health needs, access to services, and effectiveness of MHS delivery to the nearly two billion Muslims globally is greatly needed. Clinicians and scholars from Muslim-majority countries may be in a better position to understand the social, cultural, and regional context of their respective populations; however, based on our analysis they are greatly under-represented in the literature.

## Conclusion

Several important steps are being taken to improve the representation of the global mental health literature. The WHO (through programs such as Mental Health Global Action Program) trains and develops mental health professionals and stakeholders from low- and middle-income (LAMI) countries in research methods, program development, and policy implementation (Chisholm *et al*. 2007). Furthermore, academic institutions (such as the Harvard Global Mental Health: Trauma and Recovery Certificate Program and Johns Hopkins Global Mental Health Program) train and develop international researchers (Thornicroft *et al*. 2012). Several organizations as well as publishers have ensured researchers from LAMI countries have access to the scientific literature (Kobeisy, 2004). Finally, journals such as Lancet Psychiatry have programs to recruit and develop global mental health experts as editors in the field.

In spite of all the efforts to promote mental health research in LAMI countries, our bibliometric analysis demonstrates relatively low research output in Muslim-majority countries and very limited collaborations across regions. While MMH research may be shaped by regional preferences and specific author interests, young and emerging researchers from the region should actively seek to collaborate with established researchers from North America and Western Europe, take advantage of Massive Online Open Courses (MOOCs) to develop their research methods and writing skills, and take advantage of professional development opportunities offered by universities, professional organizations, and journals.

## Appendix 1.

Muslim majority country stem terms included: ‘Morocc’ or ‘Afghanistan’ or ‘Tunisia’ or ‘Iran’ or ‘Western Sahara’ or ‘Mauritania’ or ‘Tajikistan’ or ‘Yemen’ or ‘Iraq’ or ‘Jordan’ or ‘Mayotte’ or ‘Somalia’ or ‘Turk’ or ‘Azerbaijan’ or ‘Maldiv’ or ‘Comoros’ or ‘Niger’ or ‘Algeria’ or ‘Palestin’ or ‘Saudi Arabia’ or ‘Djibouti’ or ‘Libya’ or ‘Uzbekistan’ or ‘Senegal’ or ‘Gambia’ or ‘Egypt’ or ‘Kyrgyzstan’ or ‘Kosovo’ or ‘Turkmenistan’ or ‘Syria’ or ‘Bangladesh’ or ‘Mali’ or ‘Indonesia’ or ‘Oman’ or ‘Kuwait’ or ‘Guinea’ or ‘Albania’ or ‘Bahrain’ or ‘Qatar’ or ‘United Arab Emirat’ or ‘Sierra Leon’ or ‘Sudan’ or ‘Malaysia’ or ‘Leban’ or ‘Burkina Faso’ or ‘Kazakhstan’ or ‘Chad’ or ‘Brunei’ or ‘Nigeria’.
